# Honey bee immune response to trace concentrations of clothianidin goes beyond the macronutrients found in artificial diets

**DOI:** 10.1038/s41598-025-94647-1

**Published:** 2025-03-28

**Authors:** Pierre W. Lau, Giovanni Tundo, Joel Caren, Weiqiang Zhang, Yu Cheng Zhu

**Affiliations:** USDA-ARS Pollinator Health in Southern Crop Ecosystems Research Unit, Stoneville, MS USA

**Keywords:** Bees, Pesticide, Nutrition, Pollen, Macronutrients, Neonicotinoids, Clothianidin, Agriculture, Agroecosystem, Runoff, Detoxification, Chemical ecology, Ecology, Immunology

## Abstract

Honey bees (*Apis mellifera*) often encounter a variety of stressors in their environment, including poor nutrition and pesticides. These stressors interact and can be exacerbated in large-scale agroecosystems. We investigated how diets varying in macronutrient ratios can affect nurse bee susceptibility to pesticide stressors. Nurse bees were fed trace concentrations of clothianidin (CLO), a neonicotinoid insecticide known to have sublethal and lethal effects on honey bees, after newly emerged bees were given diets varying in proteins and lipids, a natural pollen diet, or sucrose solution diet. Bees given pollen had improved longevity, physiology, enzyme activity, and gene expression related to pesticide detoxification. The artificial diets helped improve bee health and physiology but did little to promote bee detoxification enzymes and genes. There was no effect of the trace CLO treatments on its own, but there was an interactive effect between our higher CLO treatment and poor nutrition on bee longevity and vitellogenin expression. Our results suggest that (1) exposure to even trace concentrations of CLO can interact with poor nutrition to undermine adult bee health and (2) macronutrients in artificial diets can help promote bee physiology, but other nutrients in pollen, such as potentially phytochemicals, are more directly linked honey bee tolerance to pesticide stress.

**Supplementary Information:**

The online version contains supplementary material available at 10.1038/s41598-025-94647-1.

## Introduction

Nutrition is a multi-dimensional challenge, requiring animals to regulate the intake of multiple nutrients simultaneously, which is a key factor for an organism’s fitness and reproduction^1,2^. An individual’s nutritional needs can also depend on its physiological state. For example, honey bee (*Apis mellifera*) worker food preferences shift from a pollen and protein biased diet as young nurse bees to feed the developing brood to a carbohydrate heavy diet to fuel flight activity as forager bees^3^. An organism’s foraging preferences can also change when they are affected by a stressor. Fire ants shifted their diets to carbohydrate rich foods and reduced their lipid intake when infected with a viral pathogen, suggesting that macronutrient balance can have downstream effects on how effective an organism can tolerate a stressor^4^. These interactive effects between nutrition and stressors create synergistic effects that can have positive and negative implications on animal health. Poor nutrition can either exacerbate the effects of a stressor or high-quality nutrition can help reduce susceptibility to stressors.

In the context of honey bee health, resource availability and quality have important implications for colony health, the pollination services honey bees provide, and our agricultural food security^5^. Most of the pollination services we depend on comes from pollinators, particularly honey bees^6,7^. However, beekeepers continue to report unsustainable colony losses, such as 51% in 2020–2021 and 39% in 2021–2022^8^. Major drivers of these losses include pests, pathogens, pesticides, nutrition, and beekeeper management practices^9^. These individual stressors interact and act in concert to cause both managed honey bee and wild bee declines^10–12^.

The demand for pesticide applications to control various pests in modern large-scale agricultural systems creates an environment of high agrochemical use with multiple routes of exposure to pollinators^13^. As a result, honey bees are exposed to multiple pesticides that can additively and synergistically affect colony health^14^. Of these pesticides, neonicotinoids represent a class of systemic insecticides widely used in all major field crops in the mid-southern region, including cotton, corn, soybeans, and wheat^15^. Some neonicotinoids, such as imidacloprid, thiamethoxam, and clothianidin (CLO), are ubiquitously used as seed treatments. Consequently, residues can be detected in the soil, plant tissue, nearby wildflowers, and floral resources (pollen and nectar)^15^. Their physiochemical properties allows them to readily translocate to different parts of the plant. As a result, CLO is easily taken up by plants and expressed in bee forage^16^. On treated crops, there were an average of 1.9 ng/g CLO on corn pollen and 0.6 ng/g CLO in canola nectar^17^. These numbers are concerning, as the oral LD50 of CLO is 3.28 ng/bee after 72 h while the LC50 is approximately 25.4 ng/bee^18^. Even sublethal concentrations can reduce the weights of newly emerged bees and cause changes in their behavior and gene expression^19^. In dead-out colonies in agricultural settings, CLO was one of the two neonicotinoids detected in all pollen samples analyzed from those colonies^20^.

Understanding even low and trace levels of exposure is essential given the widespread use of neonicotinoids in agriculture, their impacts on bee health, assimilate in plant tissue, and their ability to drift to nearby systems. In some scenarios, neonicotinoid concentrations in floral resources are low, possibly partly due to their high solubility in water making them susceptible to leaching and runoff^21–23^. Leaching and runoff create additional possible routes of pesticide exposure in soil and water, which is concerning as wild bees nest in soil and pollinators seem to prefer collecting agricultural water runoff containing salts^13,24,25^. As a result of these processes, CLO was detected in 85% of aquatic wetlands sampled^26^. It is not uncommon for neonicotinoids to exceed regulatory thresholds and be detected in drinking water at 0.24 to 57.3 ng/L after maize and soy planting^21,27^. Even when CLO was only detected at trace levels below the United States Environmental Protection Agency’s chronic toxicity benchmarks, there was still a significant decline in aquatic invertebrate biomass in wetland environments^26^. It only takes 0.3 ng CLO per bee to cause sublethal effects on learning and memory^28,29^.

The heavily modified landscape also creates a nutritionally challenging environment for pollinators by limiting the abundance and diversity of resources available for bees^30,31^. There can be extreme spatial and temporal fluctuations of floral resources when a significant part of the landscape is developed and dedicated to a few crops^32–34^. The nutrients from the pollen and nectar provided by agricultural crops may also not fulfill honey bee nutritional needs^35^. However, many crops still need pollination services provided by honey bee colonies^36^. Placing bee colonies in these environments can therefore be challenging due to the combined nutritional stress and increased exposure to agrochemicals^37^.

Despite the strenuous landscape agricultural ecosystems and its impact on overall honey bee health, there are potential opportunities to mitigate the effects of pesticide exposure to bee health through improving overall bee nutrition^38,39^. From a landscape level, having supplemental forage available adjacent to croplands can provide more floral rewards and can ameliorate the negative impacts of pesticides on pollinators^40–44^. The positive bee response to floral resource supplementation in the landscape can be partly attributed to the nutritional resources upregulating and activating bee immune response to the pesticide(s), reducing individual susceptibility to the toxins they encounter^45^. At an individual level, access to high-quality pollen promotes bee development, longevity, and detoxification ability when honey bees were exposed to field relevant doses of pesticides^38,46,47^. At acute pesticide exposure levels, pollen intake can reduce the toxicity of certain pesticides^45^. However, pollen is a highly complex nutritional resource^48^, and there are many possibilities to how pollen can act as a medicinal agent towards reducing bee susceptibility to pesticides. It is possible that pollen protein content can play a substantial role in promoting pesticide metabolization^45^. The lipid fraction of pollen has also been linked to increase bee detoxification gene expression and *Defensin-1* compared to bees fed the protein fraction of the diet^39^. Finally, phytochemicals have been shown to upregulate pesticide detoxifying enzymes, such as cytochrome P450s^49,50^.

In our study, we tested the effect of dietary macronutrients on honey bees exposed to trace levels of a commonly used neonicotinoid pesticide. We used artificial diets varying in protein (P) to lipid (L) ratios (P: L), a no diet negative control, and a natural pollen diet positive control to determine if there were interactive effects between nutrition and trace doses of clothianidin (CLO). The trace concentrations simulate real-world scenarios where nurse bees would be orally exposed to CLO even if the exposure is coming from non-point sources, such as water, or if pesticide-laced nectar is diluted by bee foraging from other resources or beekeeper supplemental sucrose feeding. We hypothesized nutrition quality will interact and help reduce bee susceptibility to trace concentrations of CLO. In particular, the natural pollen diet has a similar P: L (between 1:1 to 2:1 P: L) to what honey bees were estimated to preferentially collect^51^. We also hypothesized that the balanced 30:20 artificial diet would promote markers tied to bee health and pesticide detoxification for reducing bee susceptibility to CLO.

## Materials and methods

### Honey bees

Colonies were sourced from local queen breeders from the region. Frames of sealed brood were collected from eight honey bee colonies previously established and permitted in the Mississippi Wildlife Management Area. The brood frames were randomly mixed and placed in an incubator at 33 ± 0.5 ℃ and 65 ± 3% relative humidity. Newly emerged bees were randomly transferred to cages daily, and caged bees were maintained under the same incubator conditions as previously described.

### Diets

Three artificial diets varying in protein to lipid ratio (P: L) were made based on the diets used in Powell^52^. Briefly, the diets consisted of (1) isolated soy powder (NOW Foods Sports Nutrition), (2) linseed oil (MP biomedicals), (3) 50% (w/v) sucrose, (4) Vanderzant vitamin mixture for insects (MP Biomedicals), and (5) alpha-cellulose (Sigma-Aldrich). Isolated soy powder was chosen as the protein source because its amino acid profile is balanced relative to the nutritional needs of honey bees. Linseed oil was chosen as the lipid source because of its high proportion of relevant fatty acids and its high omega-3-to-omega-6 fatty acid ratio. Diets were created by calculating the percentage of each nutrient from the total mass of the diet. The diets varied in the P: L ratio based on the pollen honey bees collect in their environment (30P:20 L)^51^, a low protein high lipid diet (20P:30 L), and a high protein diet (40P:10 L). We also have a negative control group where bees were not given a diet and a positive control group where bees were provided with natural pollen. The natural pollen is a polyfloral blend of commercial bee collected granules blended to a powder in a coffee grinder. Nutritional analyses, using methods from Lau, Lesne^48^, determined the pollen blend contained 21% protein and 19% lipid (1.1P:1 L). Upon microscopic inspection, the pollen sample used had a significant amount of pollen from the Asteraceae family, which was noted to have a low P: L ratio of ~ 1.06P:1L^51^. All cages were provided with 50% (w/v) sucrose and water in gravity feeders ad libitum.

### Pesticide

Three treatment groups, including 0.005 µg/L CLO, 0.020 µg/L CLO, and a negative control were administered to bees orally through the sucrose provided in each cage. These concentrations are based on benchmarks on acute and chronic toxicity levels in water^53–55^. The CLO was sourced from the commercial pesticide formulations (Belay 50 WDG, Valent) to include possible synergistic effects with the inactive ingredients and adjuvants^18,56^. This is more representative to field exposure than testing only the active ingredient alone.

### Caged nurse bee bioassays and assessments

Twenty newly emerged one-day-old bees were transferred to cages and provided with 50% (w/v) sucrose and water ad-libitum through a gravity feeder. We tested newly emerged bees because nurse bees represent the age group where honey bees consume the most pollen to develop their hypopharyngeal glands to feed developing larvae. Each cohort of bees was provided with their respective treatment diet of 40P:10 L, 30P:10 L, 20P:30 L, natural pollen, or no protein diet control after the first day. At this point, cages which exceeded 25% bee mortality were removed from the study. After seven days, sucrose solutions were replaced with adulterated sucrose containing 0.005 µg/L CLO, 0.020 µg/L CLO, or an unadulterated sucrose solution. Dead bees were counted and removed daily. Honey bees incapable of righting themselves were recorded as dead. Three bees in each treatment group were subsampled at day 14. Bees were dissected and analyzed for their physiological markers (hypopharyngeal gland (HPG) size, abdominal lipid, glycogen, and protein content), enzyme activity (glutathione S-transferase (GST), esterase (EST), invertase, acetylcholinesterase (AChE), and phenoloxidase (PO)), and gene expression (P450 genes and vitellogenin (vg)) related to nutrition, health, and pesticide detoxification (See detailed methods in supplementary file 1)^18,39,51,57–62^.

For the physiological markers, bee HPG size was measured following Corby-Harris and Snyder (2018), and bee abdomens were dissected and freeze dried and then analyzed following an adapted version of Foray et al. (2012) and Schneider et al. (2021). Enzyme activity was measured following Zhu et al. (2017), except that bee heads and abdomens were used instead of heads and thoraces and the absolute value of the activity was used instead of the specific activity. To measure representative p450 transcript levels, one bee abdomen per sample (three samples per treatment) was homogenized using Benchmark Scientific pre-filled tube kits (cat #D1032-30) (containing sterile, nuclease-free 3.0 mm zircon beads) and total RNA was extracted using BioRad Aurum™ Total RNA mini-kit (cat #732–6820). RNA was immediately reverse-transcribed using Thermo Scientific Verso cDNA Synthesis kit (cat #AB-1453/B), and qPCR was run using Applied Biosystems PowerUp™ SYBR™ Green Master Mix (cat #A25742). Five P450 genes, selected to represent the important detoxification activity of the CYP families, were analyzed: *Cyp6A13*, *Cyp6Aq1*, Cyp9q1, Cyp9q2, and Cyp9q3; RP-49 was used as housekeeping reference. Data were processed using the 2-ddCt method.

### Statistics

The study design was factorial, combining three pesticide treatments and five dietary treatments conducted over two consecutive trials in June and July. In total, there were 162 cages involving 3232 bees. Bee mortality was assessed using a Kaplan–Meier survival analysis and a Cox proportional hazards model to determine an individual’s risk based on the treatments. The survivorship data is right censored for the subsampled day 14 bees in each cage. Bee assessment data were checked for normality and were either log or square root transformed, if needed, for a linear mixed model (LMM) to assess the interactions between diet and pesticide exposure on bee fitness parameters. The LMM was chosen, as sample sizes for different parameters measured across diet and pesticide treatment effects were not equal. A Bonferroni correction using 28 total comparisons (10 diet + 3 pesticide + 15 interaction comparisons). Diet and pesticide exposure were incorporated in the model as fixed effects, and cage number nested within bee ID was incorporated as a random effect. Post-hoc multiple comparisons were done using a Tukey’s honestly significant difference test. In total, our bee assessments included six bees from each treatment group for our day 14 subsample and 12 bees from the end of the experiment.

## Results

### Survivorship

**Fig. 1:**
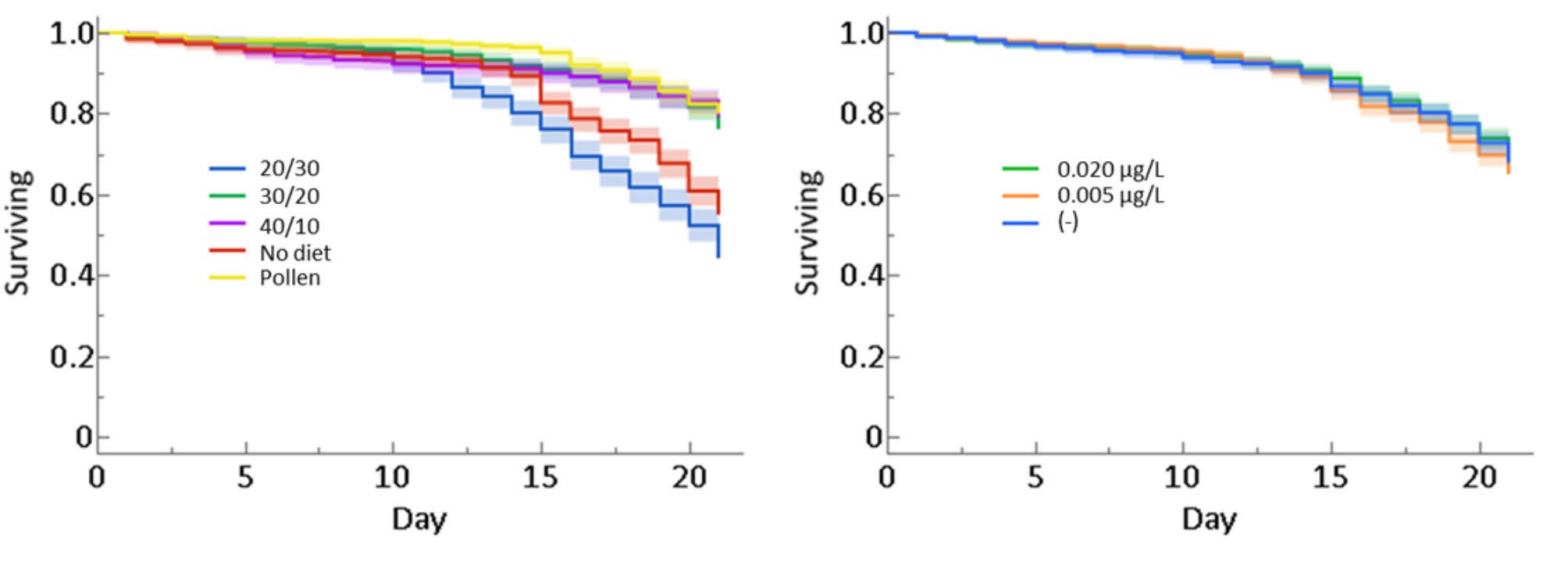
The effect of (**a**) diet and (**b**) pesticide treatment on honey bee worker longevity represented by the proportion of surviving bees. Shaded regions correspond to the 95% simultaneous confidence interval for each treatment group. See supplementary Tables 1 and 2 for the hazard ratios for each diet.

**Fig. 2:**
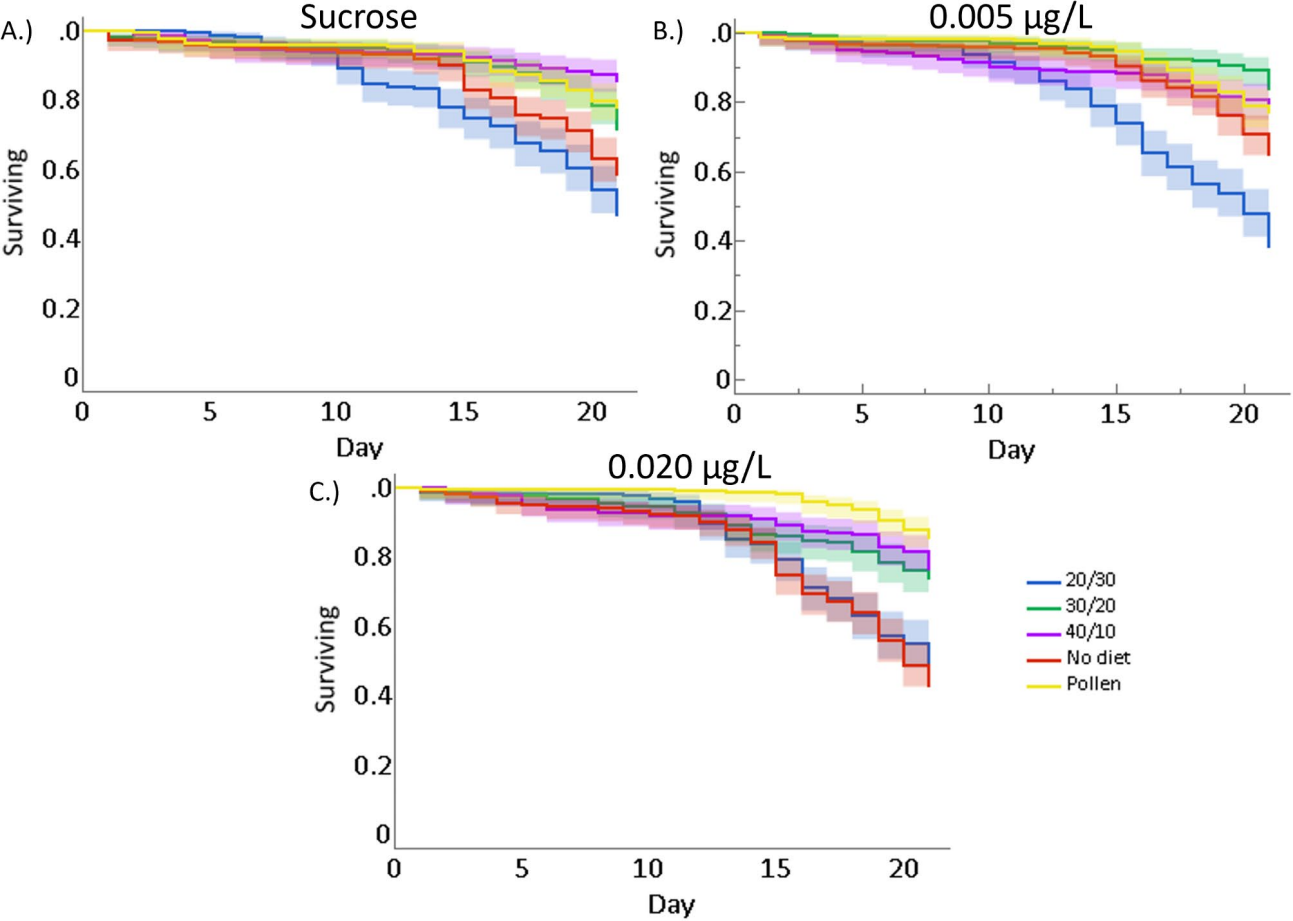
The proportion of surviving bees given different diets when treated with (**a**) sucrose control, (**b**) 0.005 µg/L CLO, and (**c**) 0.020 µg/L CLO. Shaded regions correspond to the 95% simultaneous confidence interval for each diet treatment group. See supplementary Tables 3–5 for the hazard ratios for each diet.

Worker bees given the pollen, 40:10, and 30:20 diets had significantly higher survivorship than bees given 20:30 and no diet (Cox proportional hazards model, *p* < 0.001; Fig. 1a). Worker bees given either 20:30 or no diet had between a 1.8–3.6x increased likeliness of mortality compared to bees given pollen, 40:10, or 30:20 diets (Table S1). The trace concentrations used in this experiment had no significant effect on bee survivorship (Cox proportional hazards model, *p* = 0.49; Fig. 1b). However, there was an interactive effect between diet and pesticide treatment on bee mortality (Cox proportional hazards model, *p* < 0.001; Fig. 2), implying that the trace pesticide treatments alone did not influence bee longevity, but did when combined with poor nutrition.

### Honey bee physiology

**Fig. 3:**
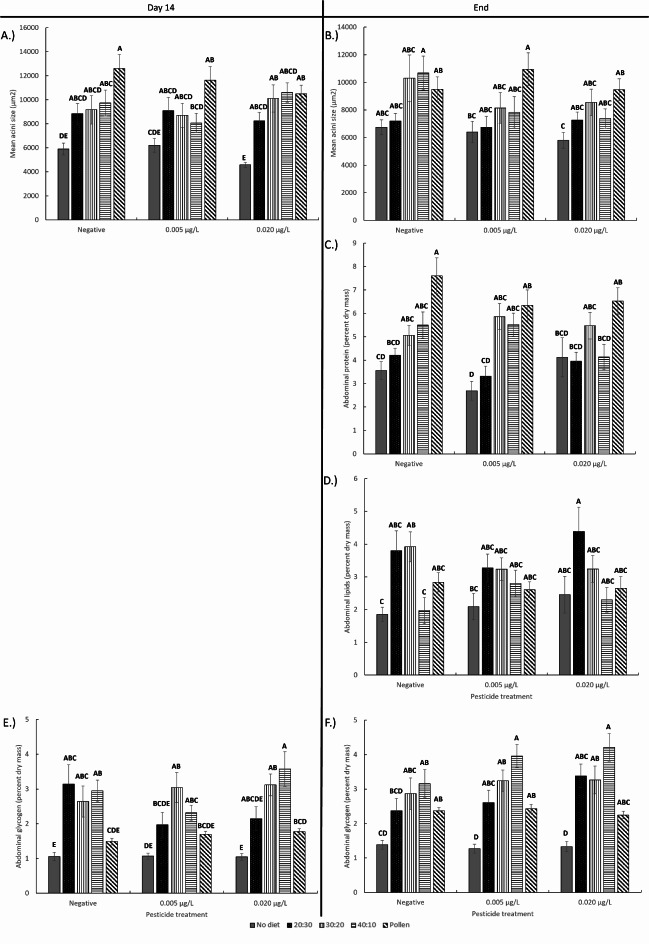
Mean honey bee (**a**,**b**) HPG acini size, (**c**) abdominal protein, (**d**) abdominal lipid, and (**e**,**f**) abdominal glycogen content at day 14 and at the end point of the study after given different diets and pesticide treatments. Means for the interactive effect followed by the same letter are not statistically significant (Tukey HSD, *p* < 0.05).

Diet significantly affected honey bee HPG sizes, abdominal protein, lipid, and glycogen content at both day 14 and the end point of the experiment (Fig. 3; Table 1; linear mixed effects model, Day 14: Logworth = 15.5, *p* < 0.001, End: Logworth = 5.9, *p* < 0.001). Bees given pollen had larger HPG sizes and higher abdominal protein (Logworth = 11.1, *p* < 0.001) content compared to bees in other diet treatment groups but having access to artificial diets still improved HPG size and abdominal protein content compared to bees having no access to diet at all (Table S6). In contrast, bees given the higher lipid diets, 20:30 and 30:20, had significantly higher abdominal lipid levels compared to bees given no protein diet and the 40:10 artificial diet (Logworth = 5.4, *p* < 0.001). Abdominal glycogen levels were also higher in bees given the artificial diets compared to bees given pollen or the no protein diet (Day 14: Logworth = 21.7, *p* < 0.001, End: Logworth = 19.5, *p* < 0.001). There were no pesticide and interactive effects between pesticide and diet on honey bee physiology.

**Table 1:** Output of linear mixed models conducted on honey bee physiology parameters in response to pesticide, diet, and pesticide*diet fixed model effects. Significant values are in bold.

Variable	Period	Factor	DF	F Ratio	*P*-value
HPG	14	Pesticide	2	0.53	0.59
		**Diet**	**4**	**23.4**	< 0.001*
		Pesticide*Diet	8	1.6	0.13
	End	Pesticide	2	2.3	0.10
		**Diet**	**4**	**8.7**	< 0.001*
		Pesticide*Diet	8	0.97	0.46
Protein	End	Pesticide	2	1.06	0.33
		**Diet**	**4**	**16.3**	< 0.001*
		Pesticide*Diet	8	1.52	0.15
Lipid	End	Pesticide	2	0.07	0.93
		**Diet**	**4**	**8.08**	< 0.001*
		Pesticide*Diet	8	0.83	0.58
Glycogen	14	Pesticide	2	1.42	0.24
		**Diet**	**4**	**34.5**	< 0.001*
		Pesticide*Diet	8	1.71	0.10
	End	Pesticide	2	2.87	0.06
		**Diet**	**4**	**29.8**	< 0.001*
		Pesticide*Diet	8	1.4	0.20

### Enzyme activity

**Fig. 4:**
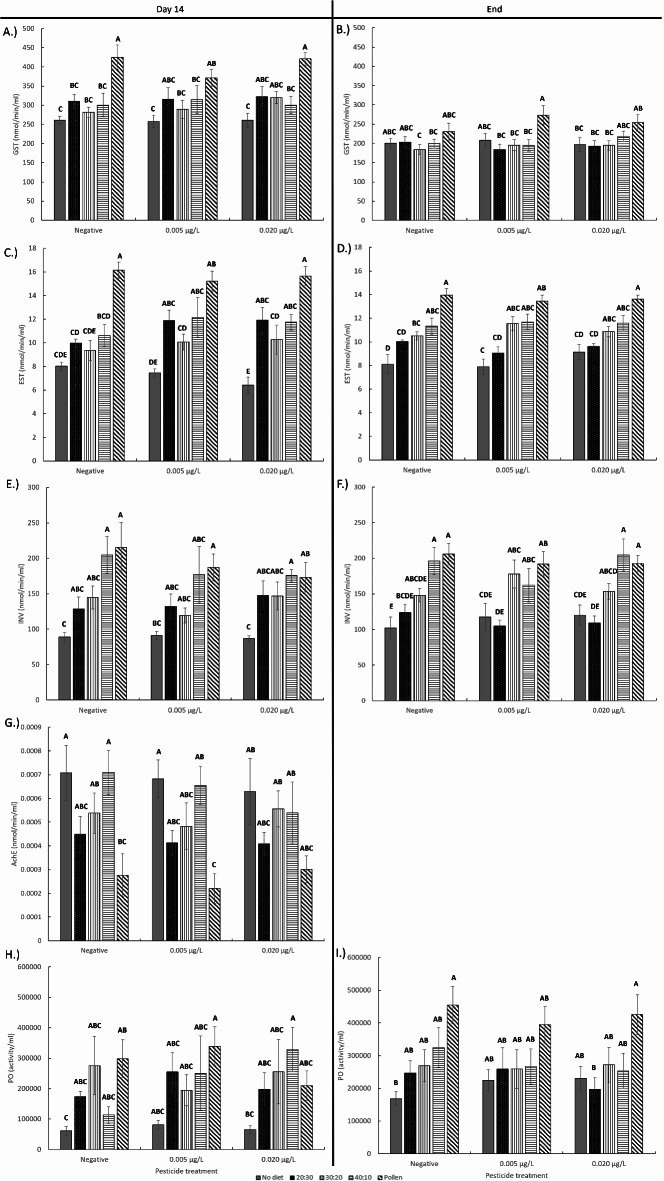
Mean honey bee (**a**,**b**) GST, (**c**,**d**) EST, (**e**,**f**) INV, and (**g**) AchE, and (**h**,**i**) PO activity levels at day 14 and at the end point of the study after given different diets and pesticide treatments. Means for the interactive effect followed by the same letter are not statistically significant (Tukey HSD, *p* < 0.05).

Each of the honey bee enzymes analyzed were significantly affected by the diet they were given at both day 14 and the end of the study (Fig. 4; Table 2; linear mixed effects model, *p* < 0.001). At day 14, bees given pollen had significantly higher activity levels of GST, EST, and INV. Honey bees given artificial diets also had higher GST, EST, and INV activity levels compared to the no diet treatment bees, but lower activity compared to the pollen-fed bees (Tukey HSD, *p* < 0.05; Supplementary Table 6). In pollen-fed bees, AChE activity was significantly lower compared to bees fed the other dietary treatments. Bees had similar PO enzyme activity levels when they had access to any of the artificial or pollen diets but had significantly lower PO activity levels when not given any protein diet.

**Table 2:** Output of linear mixed models conducted on honey bee enzyme activity parameters in response to pesticide, diet, and pesticide*diet fixed model effects. Asterisks denote a significant effect at *p* < 0.05. Significant values are in bold.

Variable	Period	Factor	DF	F ratio	*P*-value
GST	14	Pesticide	2	0.82	0.44
		**Diet**	**4**	**20.6**	< 0.001*
		Pesticide*Diet	8	0.52	0.84
	End	Pesticide	2	0.21	0.81
		**Diet**	**4**	**9.6**	< 0.001*
		Pesticide*Diet	8	0.81	0.59
EST	14	Pesticide	2	0.4	0.67
		**Diet**	**4**	**37.3**	< 0.001*
		Pesticide*Diet	8	1.19	0.31
	End	Pesticide	2	0.6	0.55
		**Diet**	**4**	**36.2**	< 0.001*
		Pesticide*Diet	8	1.0	0.45
INV	14	Pesticide	2	0.75	0.48
		**Diet**	**4**	**16**	< 0.001*
		Pesticide*Diet	8	0.45	0.88
	End	Pesticide	2	0.06	0.94
		**Diet**	**4**	**28.1**	< 0.001*
		Pesticide*Diet	8	0.79	0.62
AChE	14	Pesticide	2	0.19	0.83
		**Diet**	**4**	**11.8**	< 0.001*
		Pesticide*Diet	8	0.71	0.68
	End	Pesticide	2	0.81	0.45
		**Diet**	**4**	**44.9**	< 0.001*
		Pesticide*Diet	8	1.0	0.44
PO	14	Pesticide	2	0.39	0.68
		**Diet**	**4**	**8.42**	< 0.001*
		Pesticide*Diet	8	1.15	0.34
	End	Pesticide	2	0.82	0.44
		**Diet**	**4**	**8.7**	< 0.001*
		Pesticide*Diet	8	0.56	0.81

Overall, bee enzyme activity levels were mostly consistent at the end of the experiment compared to the activity levels from day 14. GST activity was significantly higher in pollen fed bees with no difference between each of the other diet groups (Tukey HSD, *p* < 0.05; Supplementary Table 7). For EST, bees had the highest activity level when fed pollen. Feeding on higher protein artificial diets (40:10 and 30:20) also significantly increased EST activity compared to the 20:30, which still had significantly higher activity compared to bees not given any protein diet. Bees had the lowest INV activity levels when given the 20:30 diet. In contrast to the day 14 PO activity levels, PO activity at the end point was only significantly higher in bees given the natural pollen diet. There were no pesticide and interactive effects between pesticide and diet on honey bee enzyme activity.

### Gene expression

**Fig. 5:**
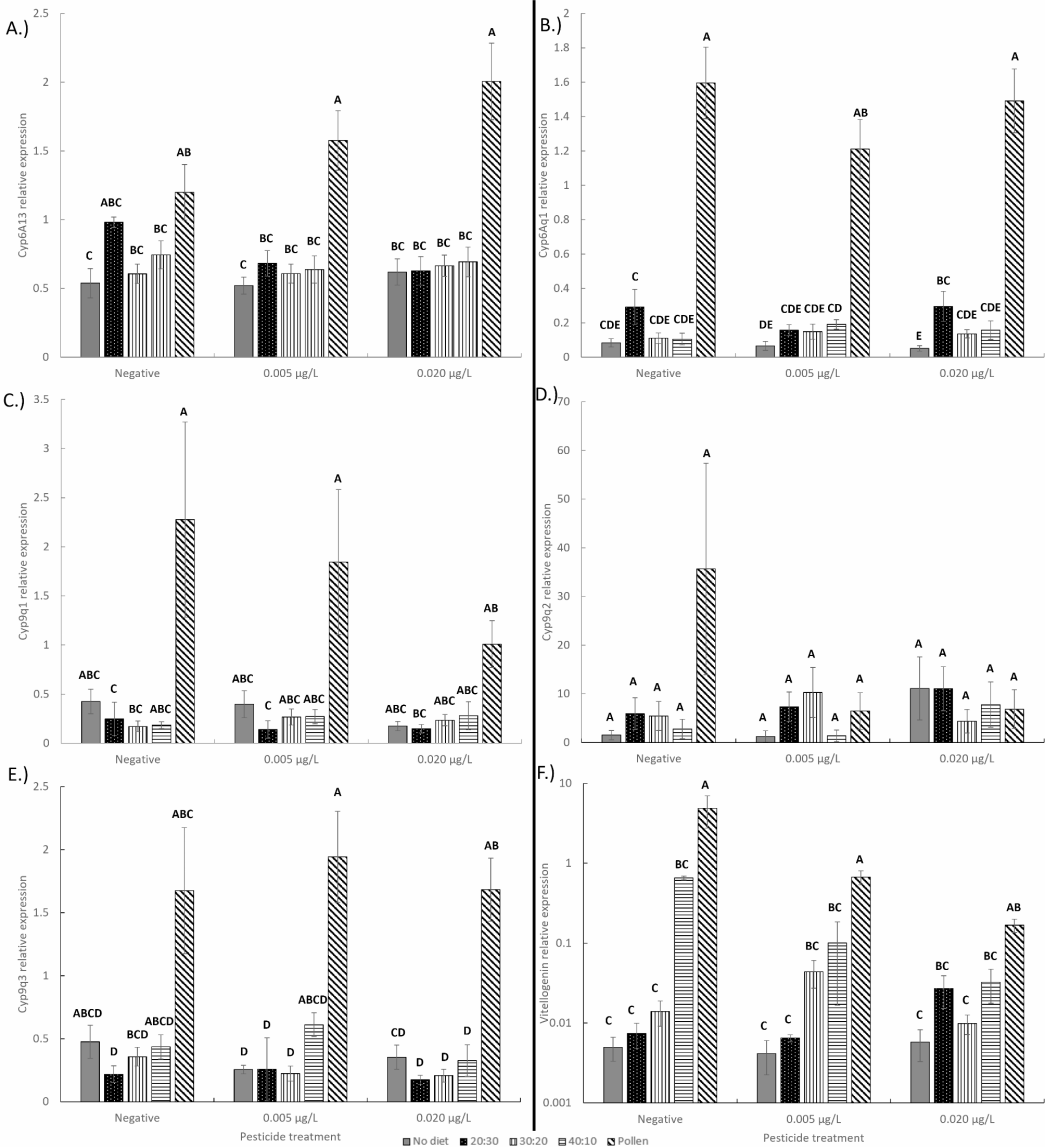
Mean honey bee (**a**) Cyp4A13, (**b**) Cyp6Aq1, (**c**) Cyp9q1, (**d**) Cyp9q2, (**e**) Cyp9q3, and (**f**) Vg expression at the end point of the study after given different diets and pesticide treatments. Means for the interactive effect followed by the same letter are not statistically significant (Tukey HSD, *p* < 0.05).

All cytochrome P450 genes analyzed in bees besides *Cyp9q2* were significantly affected by the diets they were given (Fig. 5; Table 3; linear mixed effects model, *p* < 0.001). Pollen significantly increased *Cyp6A13*, *Cyp9q1*, and *Cyp9q3* expression compared to all other dietary treatment groups (Tukey HSD, *p* < 0.05; Supplementary Table 7). Bees given pollen diets also had significantly higher vg expression levels compared to all other dietary treatment groups. The 40:10 and 30:20 artificial diets still promoted vg expression compared to bees given no protein diet at all. Artificial diets only increased *Cyp6Aq1* expression (Tukey HSD, *p* < 0.05). There were no notable dietary differences in the other P450 genes. There were also no pesticide and interactive effects between pesticide and diet on honey bee P450 gene expression. In contrast, there was an interactive effect between pesticide treatment and nutrition on honey bee vg expression. Increasing CLO exposure and diets decreasing in protein interactively lowered vg expression in bees.

**Table 3:** Output of linear mixed models conducted on honey bee P450 and Vg gene expression parameters in response to pesticide, diet, and pesticide*diet fixed model effects. Asterisks denote a significant effect at *p*<0.05. Significant values are in bold.

Variable	Period	Factor	DF	F ratio	*P*-value
*Cyp6A13*	End	Pesticide	2	0.44	0.64
		**Diet**	**4**	**22.3**	< 0.001*
		Pesticide*Diet	8	1.6	0.14
*Cyp6Aq1*	End	Pesticide	2	0.02	0.99
		**Diet**	**4**	**41.9**	< 0.001*
		Pesticide*Diet	8	1.0	0.41
*Cyp9q1*	End	Pesticide	2	0.09	0.92
		**Diet**	**4**	**12.9**	< 0.001*
		Pesticide*Diet	8	0.57	0.80
*Cyp9q2*	End	Pesticide	2	0.23	0.80
		Diet	4	1.6	0.18
		Pesticide*Diet	8	0.76	0.64
*Cyp9q3*	End	Pesticide	2	0.75	0.48
		**Diet**	**4**	**21.9**	< 0.001*
		Pesticide*Diet	8	0.74	0.66
Vg	End	Pesticide	2	0.84	0.44
		**Diet**	**4**	**40.8**	< 0.001*
		Pesticide*Diet	**8**	**2.3**	0.03*

## Discussion

Our results emphasize the importance of quality diets and nutrition for honey bees to promote individual fitness and to reduce their susceptibility to agrochemicals. We show that even trace levels of CLO can synergistically interact with poor nutrition to negatively affect bee fitness. We also demonstrated the direct effects of nutrition promoting honey bee health and improving longevity with potential to foster tolerance to trace concentrations of CLO exposure administered by feeding. To our knowledge, this study is the first to: (1) demonstrate interactive effects of trace concentrations of CLO and poor nutrition; and (2) show that macronutrients alone in artificial diets do little to promote honey bee detoxification mechanisms to mitigate the interactive negative effects of CLO exposure and nutritional stress. Instead, other properties in pollen likely play a larger role towards upregulating honey bee immune response to trace concentrations of CLO exposure.

The diets we used in this study are unique compared to previous studies on bee nutrition and pesticides. Here, we created artificial diets simulating the P: L of the pollen honey bees preferentially collect in the environment^51,52,63^. These diets were then manipulated to alter the macronutrients while keeping other nutrient ratios constant, enabling us to isolate and test the effects varying proteins and lipids in diets. The artificial diets were distinct enough for incurring effects on honey bee health, especially their longevity and physiology. However, pollen chemistry is highly complex, and our results suggests that the artificial diets used in this study are still lacking constituents that make up the multifaceted chemical nature of pollen. It should be noted that our diets lack many of the phytochemicals and sterols regularly found in pollen^64–66^. Regardless, our balanced 30P:20 L diet closely resembles the P: L ratio of the pollen used in this study (1.1P:1 L) and was comparable for promoting honey bee longevity, HPG size, abdominal protein content, abdominal lipid content, and glycogen. These physiological metrics are important for individual bee and overall colony health, as the honey bee HPG is used to produce food for the developing larvae, abdominal protein is important for protein synthesis, abdominal lipids is important for the fat body involved in vg synthesis, metabolism, energy storage, and detoxification, and glycogen is important for energy metabolism^67–69^. Similar to other studies, bees fed diets containing protein had elevated levels of crude protein in the abdomen, while bees fed diets higher in fat had higher abdominal lipid content^63,67^. The abdominal glycogen levels were highest in bees fed the artificial diets, which reflects the sucrose solution used to create the diets.

While most studies use sublethal and lethal concentrations of CLO^18,19,70,71^, we chose to use trace concentrations. This resulted in no significant effect of the pesticide treatments on caged bees. However, we observed interactive effects between the higher 0.020 µg/L concentration of CLO with poor nutrition. Honey bees given no-protein diet or the 20:30 diet had lower survivorship when exposed to 0.020 µg/L CLO compared to the other dietary treatment groups. Bees had higher survival when given natural pollen. We also only started seeing significant positive effects of survivorship between bees fed pollen compared to the 40:10 and 30:20 dietary groups in the 0.020 µg/L treatment groups. There was also an interactive effect between diet and pesticide treatment for vg expression. These differences may be more pronounced if we tested a higher dose of CLO. When honey bees were deprived of nutrition, developing workers were found to be have higher levels of oxidative stress and more susceptible to sublethal concentrations of CLO^72^. Our results with trace concentrations of CLO and poor nutrition highlight the importance of understanding how even lower concentrations of pesticides can interact with other stressors to affect bee health.

Using trace concentrations of CLO is a conservative approach for testing CLO toxicity to bees. These concentrations are below the sublethal range known to affect honey bee health and fall below the level of detection (LOD) in many commonly used methods. The USDA-APHIS National Science Laboratory in Gastonia, NC has a LOD for clothianidin using a broad screening method at 1.5 ppb^73^. The National Science Laboratory is a commonly used analytical lab for surveying pesticide residues in bee resources^74–76^. As a result, CLO is not detected in several studies surveying pesticide residues in bee resources even when colonies were in high agricultural areas^14,23,30^. This occurs despite CLO detection in soil, which was found to be comparable to CLO concentrations in corn pollen, a resource bees use in the summer months^15,17,30,77^. There may still be CLO exposure at lower levels, especially if bees collect contaminated water sources and or if a contaminated source is diluted with other non-crop resources coming in^78,79^. The proportion of crop pollen compared to non-crop pollen remains low throughout the year^30^. Honey can also be less contaminated than nectar, as honey is the sum of resources collected over an extended period of time^80^. Like pollen, there can be a dilution effect even if there is a contaminated source of nectar^79^. Both pollen and nectar have been found to contain CLO years after application^81^. Finally, CLO is found at trace concentrations in water systems bee foragers may be collecting water from. These trace concentrations have notable effects on the invertebrate community^21,26,82^. Due to the toxicity of CLO and the multiple routes of exposure, the Canadian Pest Management Regulatory Agency set their acute benchmark at 1.5 µg/L (1.5 ppb) CLO and chronic levels at 0.0015 µg/L (1.5 ppt) CLO to protect 95% of aquatic invertebrates to^53–55^. Therefore, we believe the CLO concentrations we used in our studies are representative of real-world conditions for honey bees.

Honey bees have mechanisms to metabolize insecticides^49^, including neonicotinoids, and there is evidence that dietary supplementation can support this process^83^. Oral pesticide exposure is one of the primary routes of agrochemical exposure at the colony level^13^, but nurse bees are able to create brood food secretions with little to no pesticides^84^. This suggests that nurse bees have mechanisms to metabolize pesticides and act as an environmental buffer to the developing bees they are responsible for feeding. Nurse bees are also the caste consuming the most amount of pollen to develop their HPGs and possibly to acquire the resources needed to break down pesticides^85^. In addition, honey bees are able to break down nicotine, a highly toxic alkaloid that is chemically related to neonicotinoids, with sufficient energetic resources upregulating the pathways for metabolism^86^. Members of the CYP6 and CYP9 families of cytochrome P450s are typically associated with pesticide detoxification^87,88^. Our results show that bees fed pollen diets expressed higher levels of four of the five P450 genes we tested. Pollen fed bees also had higher GST activity, an enzyme that is widely distributed in honey bee tissues^89^ and involved with pesticide resistance phase II detoxification and protection against oxidative stress^86^. Pollen also increased EST activity, which is another detoxification enzyme that is typically associated with organophosphate detoxification^90^. We only had results for AChE on day 14 bees, but bees fed pollen had reduced AChE activity. AChE is involved with deactivating the neurotransmitter, acetylcholine, and neonicotinoids mimic acetylcholine and stimulates continuous AChE production, which makes it a reliable marker to assess bee exposure to neonicotinoid insecticides^91^. It is possible that the lower AChE in pollen fed bees is associated with the increased CLO detoxification through higher P450 expression. Having access to pollen and then artificial diets also led to higher levels of INV and PO activity levels. INV is a metabolic enzyme involved with honey production while PO is important for bee immunity to pathogens^58,92^. Although these enzymes are not directly related to pesticide detoxification, it is important to understand how enzymes are affected by diets and pesticides in the context of possible synergistic effects bees would encounter in their natural environment^12,93^.

There have been several suggestions on what parts of pollen upregulate insect immune response to pesticides, and many studies point to phytochemicals as a primary factor. One way insects develop resistance to pesticides is by regulating phytochemicals and overexpressing P450s for insecticide resistance^94,95^. There is a strong correlation between pest insects, including mosquitoes, flies, planthoppers, and caterpillars, developing resistance to pesticides by overexpressing detoxification genes and enzymes, including P450s, GSTs, and ESTs^96^.

Individual phytochemicals can induce different CYP genes related to insecticide pesticide resistance, such as how caffeine can promote CYP6-like genes^97^. Specific phytochemicals supplemented in bee diets have also been shown to have varying effects depending on the concentration and context of the treatment. P-coumaric acid and kaempfereol were shown to increase HPG size while HPG size decreased when bees received caffeine or gallic acid^98^. Both caffeine and gallic acid were phytochemicals that are not regularly available in pollen throughout the season while p-coumaric acid was^99^. Phytochemical supplementation can also benefit honey bee longevity and infection with a commonly found microsporidian pathogen in honey bees^65^. However, the effects of phytochemicals can vary dependent on the type and concentration used for treatment. Phytochemical interaction with the pesticide thiamethoxam, had mixed effects, with some concentrations interacting synergistically to increase bee mortality to the pesticide^100^. A review and a table on which phytochemicals transcribe P450s is summarized in Vandenhole^101^. Pollen macronutrients have also been linked to improved bee response to pesticides. Dietary proteins were linked to influencing detoxification with phytochemicals^49^. In contrast, diets containing too much protein combined with pesticide exposure, had a detrimental effect on bee longevity while diets with a lower P: L ratio improved bee longevity^39^. Our results on how nutrition interacted with bees exposed to CLO suggested a larger effect of phytochemicals. Although the artificial diets did help promote bee physiological metrics, such as HPG, abdominal protein, lipid and glycogen content and led to similar bee longevity levels when fed 40:10 and 30:20 diets compared to bees fed pollen diets, the artificial diets did little to promote CYP genes involved with CLO detoxification and enzyme activity compared to pollen fed bees. Compared to the artificial diets, which may contain small amounts of phytochemicals from the flaxseed oil used to create the diets^102^, commercial bee pollen contain a multidimensional group of nutrients, including phytochemicals^103^, that may act to activate bee detoxification processes to pesticides. It is possible that this can explain the bee survivorship curves and how bees given that bees provided with pollen had the highest survivorship when exposed to 0.020 µg/L CLO but was lower than the survivorship of bees given the 40:10 and 30:20 artificial diets in the 0 ppb and 0.005 µg/L CLO treatment groups.

Effects on how bees digest and assimilate nutrients and how the microbiota should also be taken into consideration. Pollen is typically consumed as bee bread and predigested by bacteria^104^. The honey bee microbial community is highly robust and dominated by a set of the same bacterial species found worldwide, but disrupting the microbiome can lead to negative impacts on bee health and their susceptibility to disease^105,106^. The artificial diets used in this study was shown to affect the abundance and diversity of the bee microbiome^52^. Compared to bees fed pollen, nurse bees fed the 30:20 artificial diets reduced diversity, evenness, and even the amount of beneficial bacterial. Artificial diets were also shown to affect bee microbiota at the colony level in the field^107^. Changes to the bee microbiota can have effects on how individuals metabolize nutrients and even pesticides, like CLO^108,109^. Although we did not analyze the bee microbiome in this study, the document changes to a bee’s microbiota by feeding them artificial diets^52^ could have certainly played a role in bee susceptibility to CLO in this study.

To our knowledge, this is the first study that looked at how varying levels of proteins and lipids in artificial diets can affect bee susceptibility to trace concentrations of a commonly used neonicotinoid. Although bees were not affected by trace CLO concentrations by themselves, higher CLO interacted with bees on imbalanced diets, affecting their overall fitness. We also saw how these macronutrients by itself can improve bee health compared to not having supplemental diet at all. However, honey bees given pollen, containing a multidimensional range of nutrients, had higher expression and activity levels of the genes and enzymes linked to pesticide detoxification.Future research should precisely determine which and how nutrients, such as phytochemicals, can be applied in bee supplements to remedy pesticide exposure in bees will be critical for improving overall bee health in today’s environment^110^.

## Electronic supplementary material

Below is the link to the electronic supplementary material.

Supplementary Material 1

Supplementary Material 2

## Data Availability

The data set from this current study can be found on Ag Data Commons research repository (https://doi.org/10.15482/USDA.ADC/26026723.v1 ) and is available upon request by contacting Pierre Lau (pierre.lau@usda.gov).
