# Supplementary material for: Honey bee immune response to trace concentrations of clothianidin goes beyond the macronutrients found in artificial diets
Source: Sci Rep. 2025 Mar 28;15:10738. doi: 10.1038/s41598-025-94647-1 (PMC11953415; doi:10.1038/s41598-025-94647-1)
Supplement: Supplementary file 1 — Supplementary Material 1 [file 41598_2025_94647_MOESM1_ESM.docx]

***Supplementary file 1. Detailed methods and protocols for bee assessments***

**Protein/lipid/glycogen protocol for bees**

Purpose: Measure protein, lipid, and glycogen content in bee abdomens.

**Preparation**

Adapted from Foray et al. (2012)

1. Label 2mL screw-cap Eppendorf tubes for each bee abdomen.
2. (Optional) Dissect out the honey crop and guts.
3. Freeze dry each bee abdomen for 24 hours.
4. Weigh each individual bee and record the weights.
5. Place each bee in its own 2mL tube containing a stainless-steel bead and 360 microliters of aqueous lysis buffer solution [100 mM KH_2_PO_4_, 1 mM dithiothreitol (DTT), and 1 mM ethylenediaminetetraacetic acid (EDTA), pH 7.4].
6. Homogenize bees in a bead mill set at 4m/s for 60s, and then follow a protocol for protein content determination using Bradford.

**Protein content determination** (Bradford assay)

1. Centrifuge the samples for 5 minutes at 15000 RCF and 4°C.
2. At this point, the lipids may not be dispersed homogenously and may form a thin surface slick at the top of the liquid, so care must be used to avoid the lipids. Carefully, pipette out 10μL and place in a well to dilute it by a factor of 10 using the same aqueous lysis buffer as before (Exact quantity and dilution factor is dependent on final protein values for the specific bee samples to get a final protein reading within the range of the standards).
3. Prepare the standards using 2mg/mL bovine serum albumin with a range of 0-2mg/mL. Use the same buffer as a diluent.
4. Pipette 5μL of each standard or diluted sample in triplicate into the designated well on a 96-well plate.
5. Add 250μL of the Coomassie Plus Reagent to each well.
6. Cover the plate with foil (if using an open well plate) and place on a shaker at low speed at room temperature for 10 minutes.
7. Measure the absorbance at 595nm and determine the protein content of the unknown samples by using a standard curve.

**Preparation** (continued)

1. Add 10μL of the buffer to each sample to reach a volume of 360μL. Then, add 40 microliters of 20% sodium sulphate solution to reach a final concentration of 2% sodium sulphate.
2. Add 1500 microliters of chloroform:methanol (1:2 v/v).
3. Vigorously vortex and then centrifuge for 5 minutes at 15000 RCF and 4°C to remove the glycogen from the supernatant.
4. Pipette out the supernatant into a new, labeled 15mL centrifuge tube containing 600mL of distilled water and then vortex. The water will allow for phase separation of the chloroform:methanol. Keep the pellet in the original tube for glycogen analysis later.
5. Centrifuge the 15mL tubes for 5 minutes at 2200 RCF and then pipette out the top methanol layer into a waste beaker (note that the supernatant could be saved for carbohydrate analysis). The bottom layer is the chloroform layer with lipids which should be set out in the fume hood until the chloroform completely evaporates. At this point, follow the protocol for the sulpho-phospho-vanillin (SPV) assay.

**Lipid content determination** (SPV assay)

Adapted from Vaudo et al. (2020) and Van Handel and Day (1988)

1. To make the vanillin reagent, dissolve 600mg of vanillin 100mL of water, and then add 400mL 85% phosphoric acid. Store in the dark and discard if the solution darkens (usually stable for several months).
2. Prepare the standards by dissolving 10mg of olive oil in 10mL of chloroform (1mg:1mL oil:chloroform). Olive oil was chosen because it has a similar ratio of saturated to unsaturated fats with honeybees.
3. Add 2.5, 5, 10, 20, 50, 100, 200, 400μL of the oil solution to 15mL centrifuge tubes and then evaporate overnight in the fume hood along with the samples.
4. Once the chloroform has completely evaporated from both the standards and samples, add 200μL to each tube and then heat for 10 minutes at 90-110°C. Use the same temperature for all samples.
5. Remove from the heating block, and then add 5mL of the vanillin reagent, cap, and mix.
6. Allow reddish color to develop; this will take approximately 5 minutes and be stable up to 30 minutes.
7. Dilute the samples if necessary, and then pipette into a well plate in triplicate and measure the absorbance at 525nm. Determine lipid content using a standard curve.

**Preparation** (continued)

1. For the 2mL tubes with the bee pellet, add 800 microliters of 80% methanol, vortex, and then centrifuge for 5 minutes at 15000 RCF and 4°C.
2. Pipette out the supernatant into a waste beaker and repeat the previous step to remove carbohydrates. Then follow the protocol for the anthrone assay for glycogen.

**Glycogen content determination** (Anthrone assay)

Adapted from Foray et al. (2012) and Van Handel and Day (1988).

1. To make the anthrone reagent, mix anthrone with 70% sulfuric acid to reach a final concentration of 1.42g/L. Use this within the same day and cover with aluminum foil when not in use.
2. Prepare the standards with glycogen (from oyster) using a range of 0-200μg.
3. Add 1 mL of anthrone reagent to each tube and then heat at 90°C for 15 minutes.
4. Remove all samples from the heat block and place on ice. Vortex and centrifuge the samples for 1 minute at 10000 RCF to allow debris to fall the bottom which can be used as an alternative for using a filter tip (Schneider et al. 2021).
5. Dilute the samples with a 1:5 or 1:10 dilution using anthrone reagent if necessary.
6. Pipette 200μL in triplicate into a well plate.
7. Measure absorbance at 625nm and determine the glycogen content of the unknown samples using a standard curve.

**Enzyme Assays for Honey Bees**

**Enzyme sample extraction/preparation**

1. Make extraction buffer: 50mL 0.1M NaPO4, pH 7.2 + 150µL Triton X-100 + 500 µL 100X P.I.
2. Choose samples and set up layout for 96-well plate; run each sample in triplicate and include negative controls (extraction buffer only, no sample).
3. Transfer three bee heads and abdomens to a labeled 2 ml screw-cap microcentrifuge tube containing 2 metal beads (5/32`` Grinding balls) and 2 silica beads (2.3mm ZIRCONIA).
4. Add 1000µl cold Sodium Phosphate extraction buffer (0.1M pH 7.2, 0.3% Triton X-100 and protease inhibitor). Tighten lid securely.
5. Place samples in bead mill homogenizer and run 60sec. @ setting 4.0 m/s
6. Centrifuge samples for 15 min at 20817 RCF, 4°C.
7. Transfer 700ul of supernatant to a new, labeled 1.5ml tube and centrifuge again 15 min at 14,000rpm, 4°C, to remove debris.
8. Transfer 300µl of each supernatant to its own well in a 2mL deep-well plate containing 900µl extraction buffer (1/4 dilution). Mix well by pipetting up and down 12 times. This is used for GST, INV, PO and AchE assays.
9. Transfer 100µl from each well to a corresponding well containing 400µl extraction buffer (1/20 dilution). Mix well by carefully pipetting up and down 12 times. This is used for EST.

**Esterase standard curve using α-Naphthol**

- 1. Dissolve 10.2mg of α-Naphthol in 5ml acetone (2µg/µl). Dilute this solution with 0.1M phosphate buffer (pH7.5) by mixing 0.15ml of α-Naphthol and 14.85ml buffer (0.02µg/µl).
  2. Prepare fast blue B solution by dissolving 30mg fast blue B (Tetrazotized O-dianisidine) in 10ml of 5% sodium lauryl sulphate (SDS) solution.
  3. Prepare serial concentration of α-Naphthol as below:

| α-Naphthol (µg) | 0 | 0.2 | 0.6 | 1.0 | 1.4 | 1.8 | 2.2 | 2.6 |
| --- | --- | --- | --- | --- | --- | --- | --- | --- |
| Buffer, 0.3% Triton (µl) | 15 | 15 | 15 | 15 | 15 | 15 | 15 | 15 |
| α-Naphthol, 0.02µg/µl (µl) | 0 | 10 | 30 | 50 | 70 | 90 | 110 | 130 |
| Buffer, 1% acetone (µl) | 135 | 125 | 105 | 85 | 65 | 45 | 25 | 5 |
| Total (µl) | 150 | 150 | 150 | 150 | 150 | 150 | 150 | 150 |

- 1. Add 50 µl fast blue solutions into each well of flat bottom microplate and allow development of color for 15min at room temp. Use five rep of each concentration of α-Naphthol.
  2. Determine optical density at 600nm with microplate reader
  3. Use linear regression to establish a standard curve by plotting the amounts of (µg) α-Naphthol (Y-axis) against their corresponding optical densities (O.D.) (X-axis) after subtracting the control O.D. Provide the intercept, slope and correlation coefficient of the curve.

**Esterase assay**

1. Make the stock solutions:
   1. 30mM α-Naphthyl Acetate: in a glass vial, add 11.17mg α-Naphthyl Acetate (in -20°C) to 2ml acetone; store at -20°C for up to several weeks.
   2. 0.3mM α-Naphthyl Acetate substrate solution: add 0.15ml 30mM α-Naphthyl Acetate (stored at -20°C) to 14.85ml of 0.1M Sodium Phosphate buffer, pH 7.5;
   3. Fast Blue stock solution: 30mg Fast Blue B salt (stored at 4°C) in 10mL 5% SDS; place tube on shaker and shake until dissolved, 30+ min.
2. Transfer 15µl **diluted** sample to designated wells in a clear assay plate (three reps of each treatment).
3. Add 135µl of 0.3mM α-Naphthyl Acetate to each well.
4. Vortex 1 min. to mix, unless using shaking incubator.
5. Cover plate with foil and incubate for 30min at 37°C.
6. Add 50µl Fast Blue solution to each well to stop reaction. Re-cover plate with foil.
7. Place plate on shaker 15min at room temperature.
8. Remove foil, then place plate in plate reader.
9. Use program Esterase to shake 10sec, then determine absorbance at 600nm, 30°C.
10. Calculate the amount of hydrolytic product (α-Naphthol) of general esterases for each insect by using the α-Naphthol regression equation. The amount of α-Naphthyl produced by each well can be expressed as µg.
11. Calculate activity of general esterases for each insect by using the following formula:

*( )nmol/min/mL= Net Activity x slope of std curve / α-Naphthol M.W. / sample volume (mL) / 30 mins x 1000

**Glutathione S-Transferase (GST) Assay**

Using 1-Chloro-2,4-Dinitrobenzene (CDNB) as substrate to detect GST activity (total reaction volume=200µl, final conc. of GSH=10mM; CDNB=2mM, Reaction buffer: potassium phosphate buffer pH6.5)

1. Make the stock solutions:
   1. 20mM GSH: dissolve 61.5mg L-Glutathione, reduced (stored at 4°C) in 10mL 0.1M Potassium Phosphate buffer (pH6.5 with 1mM EDTA). **make fresh each day and keep at room temperature**.
   2. 40mM CDNB: dissolve 20.25mg CDNB (stored at room temp.) in 2.5ml 95% (833uL x 3) ethanol. Mix by vortexing or shaking. **Make fresh each day**.
2. Add 80µl 0.1M Potassium Phosphate buffer, pH6.5, with 1mM EDTA to each well in a clear assay plate.
3. Add 10µl undiluted sample to each corresponding well (run in triplicate).
4. Add 100µl of 20mM GSH into each well.
5. Add 10µl of 40mM CDNB into each well, then mix by vortexing: 1min at speed=3.
6. Immediately load plate into plate reader; shake 5 seconds, then measure absorbance change over time at 340nm and 30°C for 10min with 20sec interval. Program: GST.
7. Calculate GST activity for substrate CDNB. Express enzyme activity as nmol CDNB conjugated/min/mg protein using the extinction coefficient of 5.3mM^-1^

| GST activity= | Δabs/min | × | 0.2ml | ×1000= ( )nmol/min/ml |
| --- | --- | --- | --- | --- |
|  | 5.3 mM^-1^cm^-1^ |  | 0.01ml |  |

**Acetylcholinesterase assay**

Detect AchE activity in honey bee by using Acetylthiocholine iodide (ATC) as substrate. AchE hydrolyzes ATC to yield acetate and thiocholine, and latter reacts with 5,5`-dithio-bis-2-nitrobenzoic acid (DTNB) to form a yellow product with can be measured colorimetrically at 405nm.

Final conc. of ATC (0.25mM) and DTNB (0.4mM) in Sodium Phosphate buffer pH 7.5.

1. Make stock solutions:
   1. 37.5mM ATC: in a 2.0mL tube, dissolve 15mg ATC (stored at 4°C) in 1.38ml (690uL x 2) of 0.1M sodium phosphate buffer pH 7.2 in 2.0mL tube; ATC solution is stable for several hours on ice. **Make fresh each day**.
   2. 12mM DTNB: dissolve 21.2mg DTNB (stored at room temp.) in 4.46ml of 0.1M Sodium Phosphate buffer pH 7.5. Stable for up to *two weeks at 4ºC*.
2. Make solution of ATC-DTNB reaction mixture for 1 plate (15ml, 150µL/per well) and **keep at room temperature**
   1. 133µl of 37.5mM ATC
   2. 666µl of 12mM DTNB
   3. Bring to 15ml with 0.1M Sodium Phosphate buffer pH 7.5.
3. Transfer 15µl undiluted sample to designated wells in a clear assay plate (three reps of each treatment).
4. Add 150µl ATC-DTNB reaction mixture, then vortex for 1min at speed = 3.
5. Immediately load plate on plate reader; shake 5 seconds, then read absorbance change at 412nm, 30ºC for *15min with 20sec interval*. Program: AchE.
6. Calculate AchE activity for substrate DTNB. Express enzyme activity as nmol DTNB conjugated/min/mg protein using the extinction coefficient of 1.36×10^4^

| AchE activity= | Δabs/min | × | 0.2ml | ×1000 = ( )nmol/min/l |
| --- | --- | --- | --- | --- |
|  | 1.36×10^4^ M^-1^cm^-1^ |  | 0.05ml |  |

**Phenoloxidase**

1. Make Dopamine solution:
   1. 2mM Dopamine: dissolve 8.0mg Dopamine HCl (FW=189.64, stored @ 4˚C) in 20ml 50mM sodium phosphate buffer, pH6.5 and **keep at room temperature**
2. Load 20µl undiluted enzyme into each well
3. Add 200µl Dopamine solution into each well.
4. Vortex 60sec at setting = 3.
5. Immediately measure absorbance over time at 490 nm, 30˚C using a microplate reader for 30min with 20sec interval.
6. One unit of PAP activity is defined as the amount of enzyme yielding PO that produces an increase of 0.001 absorbance units/min

**Invertase assay**

*Make solutions for Invertase enzyme assay:*

- 1. *Sodium Acetate buffer, pH4.3, 0.1M:* dissolve 3.28g Sodium Acetate anhydrous in 300mL ddH2O, then bring pH to 4.3 with glacial Acetic Acid (just a few drops); bring final volume to 400ml with ddH2O.
  2. *4-nitrophenyl-α-D-glucopyranoside* **(pNPG)***:* dissolve 20.0mg 4-nitrophenyl-α-D-glucopyranoside (stored at -20°C) in 10ml Sodium Acetate buffer (pH4.3, 0.1M). **Prepare fresh and protect from light*.***
  3. *Sodium Carbonate, pH 11.6, 0.1M:* dissolve 4.24g sodium carbonate in 400mL ddH2O*.*

1. Transfer 30µl Sodium Phosphate extraction buffer to each well in a clear assay plate (three reps of each treatment).
2. Add 10µl undiluted sample to designated wells.
3. Add 100µl pNPG.
4. Mix well by vortexing 60 sec. at speed=3 unless using shaking incubator.
5. Cover plate with aluminum foil and incubate 20 min at 30˚C.
6. Add 150µl pH 11.6 Sodium Carbonate to stop reaction.
7. Re-cover plate with aluminum foil.
8. Place plate on shaker for 3 min at room temperature.
9. Read plate at 405nm, 30˚C endpoint (unit/10ul).

**qPCR methods**

To measure representative p450 transcript levels, one bee abdomen per sample (three samples per treatment) was homogenized using Benchmark Scientific pre-filled tube kits containing sterile, nuclease-free 3.0mm zircon beads (cat #D1032-30), and total RNA was extracted using Invitrogen PureLink™ RNA mini-kits (cat #12-183-018A). RNA was immediately reverse-transcribed using Bio-Rad iScript™ Reverse Transcription Supermix (cat #1708841), and qPCR was run using Applied Biosystems PowerUp™ SYBR™ Green Master Mix (cat #A25742). Four p450 genes , selected to represent the important detoxification activity of the CYP families [refs below], were analyzed: CYP-6A13, CYP-6AQ1, CYP-9Q1, and CYP-9Q3 (see Table 1); RP-49 was used as housekeeping reference. Data were processed using the 2^-ddCt^ method.

**Table 1.** P450 primers.

| **name** | **sequence** |
| --- | --- |
| CYP-9Q1 (LOC408452) forward | CCACTCTGATAGGGTTGAAGAAG |
| CYP-9Q1 (LOC408452) reverse | TCGGTGAAGAACTTGGCTATG |
| CYP-9Q3 forward | GATGTGCGTCGAGAGTTTCC |
| CYP-9Q3 reverse | CTGTCCGGGTCGAATTTGTC |
| CYP-6AQ1 forward | AAACACACGTCAGCGGATAG |
| CYP-6AQ1 reverse | CCGAAGTAAAGACCGACGTAAG |
| CYP-6A13 (LOC727598) forward | CGGAACCTGAAGTATTCGATCC |
| CYP-6A13 (LOC727598) reverse | GATGCAATTTCTTGGCCCATC |

30:20 diet

|  | Mass (g) |
| --- | --- |
| **Amount of soy needed for 30g protein** | **36** |
| Lipids present in 36g soy | 0.75 |
| **Linseed oil needed** | **19.25** |
| Total mass of P/L | 55.25 |
| **Mass of total diet** | 110.5 |
|  |  |
| **50% sucrose w/v** | **43.6475** |
| **Cellulose** | **11.05** |
| **Vitamins** | **0.5525** |
|  |  |
| **Total** | 110.5 |
|  |  |
|  |  |

1. Zero the balance and weigh out dry ingredients (soy protein and cellulose) in a cup
2. In a separate beaker, create a 50% w/v sucrose solution using DI or molecular grade water. For three ~100g diets, you will need at least 150 ml 50% sucrose.
   1. Zero balance and weigh out 100g of sugar. Add water until it hits 200 ml to create 50% w/v
3. Weigh sucrose in a separate beaker
4. Weigh out vitamins and mix vitamins, which are water soluble, in the sucrose solution
5. Weigh out lipids
6. Combine the sucrose and lipids into the cup with the dry ingredients
7. Mix well and use the hand mixer to thoroughly mix everything together
8. Clean out all the tools used to make the diets
