# Supplementary material for: Honey bee immune response to trace concentrations of clothianidin goes beyond the macronutrients found in artificial diets
Source: Sci Rep. 2025 Mar 28;15:10738. doi: 10.1038/s41598-025-94647-1 (PMC11953415; doi:10.1038/s41598-025-94647-1)
Supplement: Supplementary file 2 — Supplementary Material 2 [file 41598_2025_94647_MOESM2_ESM.docx]

***Supplementary table 1***

| **Diet 1** | **Diet 2** | **Hazard Ratio** | **Prob>χ^2^** | **Lower 95% CI (Wald)** | **Upper 95% CI (Wald)** |
| --- | --- | --- | --- | --- | --- |
| Pollen | No diet | 2.5990955 | <.0001* | 2.1088677 | 3.2032818 |
| Pollen | 20:30 | 3.6013535 | <.0001* | 2.9295481 | 4.4272176 |
| Pollen | 30:20 | 1.2021807 | 0.1318 | 0.9461427 | 1.5275057 |
| Pollen | 40:10 | 1.0518367 | 0.6906 | 0.8201472 | 1.3489779 |
| 40:10 | No diet | 2.4710066 | <.0001* | 2.0045972 | 3.0459355 |
| 40:10 | 20:30 | 3.4238712 | <.0001* | 2.7846681 | 4.2097992 |
| 40:10 | 30:20 | 1.1429347 | 0.2745 | 0.8994092 | 1.4523975 |
| 40:10 | Pollen | 0.9507179 | 0.6906 | 0.741302 | 1.2192933 |
| 30:20 | Pollen | 0.8318217 | 0.1318 | 0.654662 | 1.056923 |
| 30:20 | 40:10 | 0.8749406 | 0.2745 | 0.6885168 | 1.1118409 |
| 30:20 | 20:30 | 2.995684 | <.0001* | 2.464223 | 3.6417656 |
| 30:20 | No diet | 2.1619841 | <.0001* | 1.7736395 | 2.635358 |
| 20:30 | Pollen | 0.2776734 | <.0001* | 0.2258755 | 0.3413496 |
| 20:30 | 40:10 | 0.2920671 | <.0001* | 0.237541 | 0.3591092 |
| 20:30 | 30:20 | 0.3338136 | <.0001* | 0.2745921 | 0.4058074 |
| 20:30 | No diet | 0.7216996 | <.0001* | 0.6174158 | 0.8435974 |
| No diet | Pollen | 0.3847492 | <.0001* | 0.3121798 | 0.4741881 |
| No diet | 40:10 | 0.4046934 | <.0001* | 0.3283064 | 0.4988533 |
| No diet | 30:20 | 0.4625381 | <.0001* | 0.3794551 | 0.5638124 |
| No diet | 20:30 | 1.385618 | <.0001* | 1.1853996 | 1.619654 |

Supplementary table 1. Cox proportional hazard ratios for bees given different diets. To interpret the hazard ratio, a bee given diet 2 has the ratio’s likeliness that the bee will die compared to a bee given diet 1. Asterisks denote a significant effect at *p*<0.05.

***Supplementary table 2***

Supplementary table 2. Cox proportional hazard ratios for bees given different concentrations of CLO-adulterated sucrose solution. To interpret the hazard ratio, a bee given pesticide 2 has the ratio’s likeliness that the bee will die compared to a bee given pesticide 1. Asterisks denote a significant effect at *p*<0.05.

| **Pesticide 1** | **Pesticide 2** | **Hazard Ratio** | **Prob>χ^2^** | **Lower 95% CI (Wald)** | **Upper 95% CI (Wald)** |
| --- | --- | --- | --- | --- | --- |
| 0 ppb | 5 ppb | 0.9827414 | 0.8366 | 0.8328916 | 1.1595516 |
| 0 ppb | 20 ppb | 1.0795052 | 0.3616 | 0.9158958 | 1.2723406 |
| 5 ppb | 0 ppb | 1.0175617 | 0.8366 | 0.8624024 | 1.2006365 |
| 5 ppb | 20 ppb | 1.0984631 | 0.2604 | 0.9327252 | 1.2936512 |
| 20 ppb | 0 ppb | 0.9263504 | 0.3616 | 0.7859531 | 1.0918273 |
| 20 ppb | 5 ppb | 0.9103629 | 0.2604 | 0.7730059 | 1.0721271 |

***Supplementary table 3***

| **Diet 1** | **Diet 2** | **Hazard Ratio** | **Prob>χ^2^** | **Lower 95% CI (Wald)** | **Upper 95% CI (Wald)** |
| --- | --- | --- | --- | --- | --- |
| Pollen | No diet | 0.478669 | <.0001* | 0.3386373 | 0.676606 |
| Pollen | 20:30 | 0.3418841 | <.0001* | 0.2437396 | 0.4795477 |
| Pollen | 30:20 | 0.7762877 | 0.1779 | 0.5370564 | 1.1220842 |
| Pollen | 40:10 | 1.5515057 | 0.0608 | 0.9802098 | 2.4557702 |
| 40:10 | No diet | 0.308519 | <.0001* | 0.2033132 | 0.4681643 |
| 40:10 | 20:30 | 0.2203563 | <.0001* | 0.1461373 | 0.3322691 |
| 40:10 | 30:20 | 0.5003447 | 0.0018* | 0.3236349 | 0.7735408 |
| 40:10 | Pollen | 0.6445352 | 0.0608 | 0.4072042 | 1.0201898 |
| 30:20 | Pollen | 1.2881823 | 0.1779 | 0.8911987 | 1.8620018 |
| 30:20 | 40:10 | 1.9986222 | 0.0018* | 1.2927566 | 3.0899016 |
| 30:20 | 20:30 | 0.440409 | <.0001* | 0.3244221 | 0.5978635 |
| 30:20 | No diet | 0.616613 | 0.0026* | 0.4503683 | 0.8442236 |
| 20:30 | Pollen | 2.9249678 | <.0001* | 2.0852985 | 4.1027396 |
| 20:30 | 40:10 | 4.5381043 | <.0001* | 3.0096091 | 6.8428789 |
| 20:30 | 30:20 | 2.2706164 | <.0001* | 1.6726226 | 3.0824042 |
| 20:30 | No diet | 1.4000915 | 0.0176* | 1.06034 | 1.8487053 |
| No diet | Pollen | 2.0891262 | <.0001* | 1.477965 | 2.9530119 |
| No diet | 40:10 | 3.2412912 | <.0001* | 2.1360025 | 4.9185191 |
| No diet | 30:20 | 1.6217629 | 0.0026* | 1.1845203 | 2.220405 |
| No diet | 20:30 | 0.714239 | 0.0176* | 0.5409191 | 0.9430937 |

Supplementary table 3. Cox proportional hazard ratios for bees given different diets in the 0ppb CLO treatment group. To interpret the hazard ratio, a bee given diet 2 has the ratio’s likeliness that the bee will die compared to a bee given diet 1. Asterisks denote a significant effect at *p*<0.05.

**Supplementary table 4**

Supplementary table 4. Cox proportional hazard ratios for bees given different diets in the 5ppb CLO treatment group. To interpret the hazard ratio, a bee given diet 2 has the ratio’s likeliness that the bee will die compared to a bee given diet 1. Asterisks denote a significant effect at *p*<0.05.

| **Diet 1** | **Diet 2** | **Hazard Ratio** | **Prob>χ^2^** | **Lower 95% CI (Wald)** | **Upper 95% CI (Wald)** |
| --- | --- | --- | --- | --- | --- |
| Pollen | No diet | 1.6288528 | 0.0077* | 1.1378109 | 2.3318124 |
| Pollen | 20:30 | 3.5516001 | <.0001* | 2.5308175 | 4.9841062 |
| Pollen | 30:20 | 0.6828705 | 0.0865 | 0.441479 | 1.0562501 |
| Pollen | 40:10 | 1.0427065 | 0.8356 | 0.7024679 | 1.5477388 |
| 40:10 | No diet | 1.5621393 | 0.0108* | 1.1084377 | 2.2015484 |
| 40:10 | 20:30 | 3.4061359 | <.0001* | 2.4676144 | 4.7016106 |
| 40:10 | 30:20 | 0.654902 | 0.05 | 0.4288772 | 1.0000453 |
| 40:10 | Pollen | 0.9590426 | 0.8356 | 0.6461039 | 1.4235525 |
| 30:20 | Pollen | 1.4644064 | 0.0865 | 0.9467455 | 2.2651136 |
| 30:20 | 40:10 | 1.5269461 | 0.05 | 0.9999547 | 2.3316699 |
| 30:20 | 20:30 | 5.200986 | <.0001* | 3.5861009 | 7.5430827 |
| 30:20 | No diet | 0.419234 | <.0001* | 0.283889 | 0.6191053 |
| 20:30 | Pollen | 0.2815632 | <.0001* | 0.2006378 | 0.3951292 |
| 20:30 | 40:10 | 0.2935878 | <.0001* | 0.2126931 | 0.4052497 |
| 20:30 | 30:20 | 0.1922712 | <.0001* | 0.1325718 | 0.2788544 |
| 20:30 | No diet | 0.4586251 | <.0001* | 0.3478677 | 0.6046464 |
| No diet | Pollen | 0.613929 | 0.0077* | 0.428851 | 0.8788807 |
| No diet | 40:10 | 0.6401478 | 0.0108* | 0.4542258 | 0.9021707 |
| No diet | 30:20 | 2.3853025 | <.0001* | 1.6152342 | 3.5225035 |
| No diet | 20:30 | 2.1804303 | <.0001* | 1.6538591 | 2.8746563 |

***Supplementary table 5***

| **Diet 1** | **Diet 2** | **Hazard Ratio** | **Prob>χ^2^** | **Lower 95% CI (Wald)** | **Upper 95% CI (Wald)** |
| --- | --- | --- | --- | --- | --- |
| Pollen | No diet | 2.0891262 | <.0001* | 1.477965 | 2.9530119 |
| Pollen | 20:30 | 2.9249678 | <.0001* | 2.0852985 | 4.1027396 |
| Pollen | 30:20 | 1.2881823 | 0.1779 | 0.8911987 | 1.8620018 |
| Pollen | 40:10 | 0.6445352 | 0.0608 | 0.4072042 | 1.0201898 |
| 40:10 | No diet | 3.2412912 | <.0001* | 2.1360025 | 4.9185191 |
| 40:10 | 20:30 | 4.5381043 | <.0001* | 3.0096091 | 6.8428789 |
| 40:10 | 30:20 | 1.9986222 | 0.0018* | 1.2927566 | 3.0899016 |
| 40:10 | Pollen | 1.5515057 | 0.0608 | 0.9802098 | 2.4557702 |
| 30:20 | Pollen | 0.7762877 | 0.1779 | 0.5370564 | 1.1220842 |
| 30:20 | 40:10 | 0.5003447 | 0.0018* | 0.3236349 | 0.7735408 |
| 30:20 | 20:30 | 2.2706164 | <.0001* | 1.6726226 | 3.0824042 |
| 30:20 | No diet | 1.6217629 | 0.0026* | 1.1845203 | 2.220405 |
| 20:30 | Pollen | 0.3418841 | <.0001* | 0.2437396 | 0.4795477 |
| 20:30 | 40:10 | 0.2203563 | <.0001* | 0.1461373 | 0.3322691 |
| 20:30 | 30:20 | 0.440409 | <.0001* | 0.3244221 | 0.5978635 |
| 20:30 | No diet | 0.714239 | 0.0176* | 0.5409191 | 0.9430937 |
| No diet | Pollen | 0.478669 | <.0001* | 0.3386373 | 0.676606 |
| No diet | 40:10 | 0.308519 | <.0001* | 0.2033132 | 0.4681643 |
| No diet | 30:20 | 0.616613 | 0.0026* | 0.4503683 | 0.8442236 |
| No diet | 20:30 | 1.4000915 | 0.0176* | 1.06034 | 1.8487053 |

Supplementary table 5. Cox proportional hazard ratios for bees given different diets in the 20ppb CLO treatment group. To interpret the hazard ratio, a bee given diet 2 has the ratio’s likeliness that the bee will die compared to a bee given diet 1. Asterisks denote a significant effect at *p*<0.05.

***Supplementary table 6***

| **Parameter** | **Diet** | **Connecting letters** | **Least Squares Mean** |
| --- | --- | --- | --- |
| HPG (mean acini size µm) | Pollen | A | 9.28 |
|  | 40:10 | B | 9.07 |
|  | 30:20 | B | 9.05 |
|  | 20:30 | B | 9.02 |
|  | No diet | C | 8.57 |
| Glycogen (percent dry mass) | 40:10 | A | 0.96 |
|  | 30:20 | A | 0.91 |
|  | 20:30 | AB | 0.68 |
|  | Pollen | B | 0.47 |
|  | No diet | C | -0.01 |
| GST (nmol/min/ml) | Pollen | A | 5.99 |
|  | 20:30 | B | 5.74 |
|  | 40:10 | B | 5.70 |
|  | 30:20 | B,C | 5.68 |
|  | No diet | C | 5.55 |
| EST (nmol/min/ml) | Pollen | A | 2.75 |
|  | 40:10 | B | 2.42 |
|  | 20:30 | B | 2.40 |
|  | 30:20 | B | 2.27 |
|  | No diet | C | 1.97 |
| INV (nmol/min/ml) | Pollen | A | 5.21 |
|  | 40:10 | A,B | 5.15 |
|  | 30:20:00 | B | 4.88 |
|  | 20:30 | B | 4.86 |
|  | No diet | C | 4.48 |
| AchE (nmol/min/ml) | No diet | A | -7.41 |
|  | 40:10 | A | -7.44 |
|  | 30:20 | A | -7.62 |
|  | 20:30 | A | -7.81 |
|  | Pollen | B | -8.45 |
| PO (nmol/min/ml) | Pollen | A | 12.42 |
|  | 20:30 | A | 12.05 |
|  | 30:20 | A | 12.04 |
|  | 40:10 | A | 11.89 |
|  | No diet | B | 11.01 |

Supplementary table 6. Connecting letters report from the Tukey HSD multiple comparisons test on how diet effected the parameters measured on day 14 honey bees. The diet order is arranged from the highest to lowest and the least squares means is on the transformed data. Different letters represent significant differences. Asterisks denote a significant effect at *p*<0.05.

***Supplementary table 7***

| **Parameter** | **Diet** | **Connecting letters** | **Least Squares Mean** |
| --- | --- | --- | --- |
| HPG (mean acini size) | Pollen | A | 9.13 |
|  | 30:20 | AB | 8.94 |
|  | 40:10 | AB | 8.94 |
|  | 20:30 | BC | 8.80 |
|  | No diet | C | 8.67 |
| Abdominal protein | Pollen | A | 2.54 |
|  | 30:20 | AB | 2.28 |
|  | 40:10 | BC | 2.16 |
|  | 20:30 | CD | 1.90 |
|  | No diet | D | 1.78 |
| Abdominal lipid (percent dry mass) | 20:30 | A | 1.85 |
|  | 30:20 | A | 1.80 |
|  | Pollen | AB | 1.60 |
|  | 40:10 | B | 1.44 |
|  | No diet | B | 1.37 |
| Glycogen (percent dry mass) | 40:10 | A | 1.22 |
|  | 30:20 | AB | 0.97 |
|  | 20:30 | B | 0.84 |
|  | Pollen | B | 0.84 |
|  | No diet | C | 0.19 |
| GST (nmol/min/ml) | Pollen | A | 5.65 |
|  | 40:10 | B | 5.45 |
|  | No diet | B | 5.42 |
|  | 20:30 | B | 5.41 |
|  | 30:20 | B | 5.37 |
| EST (nmol/min/ml) | Pollen | A | 2.61 |
|  | 40:10 | B | 2.43 |
|  | 30:20 | B | 2.38 |
|  | 20:30 | C | 2.25 |
|  | No diet | D | 2.10 |
| INV (nmol/min/ml) | Pollen | A | 5.07 |
|  | 40:10 | A | 5.00 |
|  | 30:20 | A | 4.93 |
|  | No diet | AB | 4.71 |
|  | 20:30 | B | 4.57 |
| PO (nmol/min/ml) | Pollen | A | 12.43 |
|  | No diet | AB | 11.89 |
|  | 20:30 | B | 11.61 |
|  | 30:20 | B | 11.50 |
|  | 40:10 | B | 11.36 |
| *Cyp6A13* (relative expression) | Pollen | A | 0.40 |
|  | 20:30 | B | -0.33 |
|  | 40:10 | B | -0.42 |
|  | 30:20 | B | -0.50 |
|  | No diet | B | -0.64 |
| *Cyp6Aq1* (relative expression) | Pollen | A | 0.32 |
|  | 20:30 | B | -1.62 |
|  | 40:10 | B | -2.20 |
|  | 30:20 | B | -2.27 |
|  | No diet | C | -3.05 |
| *Cyp9q1* (relative expression) | Pollen | A | 0.15 |
|  | No diet | B | -1.52 |
|  | 40:10 | BC | -1.66 |
|  | 30:20 | BC | -1.78 |
|  | 20:30 | C | -2.49 |
| *Cyp9q2* (relative expression) | Pollen | A | 0.79 |
|  | 30:20 | A | 0.56 |
|  | 20:30 | A | -0.10 |
|  | 40:10 | A | -1.09 |
|  | No diet | A | -2.32 |
| *Cyp9q3* (relative expression) | Pollen | A | 0.48 |
|  | 40:10 | B | -0.99 |
|  | ND | BC | -1.19 |
|  | 30:20 | BC | -1.56 |
|  | 20:30 | C | -1.88 |
| Vg (relative expression) | Pollen | A | -0.37 |
|  | 40:10 | B | -3.90 |
|  | 30:20 | B | -4.55 |
|  | 20:30 | BC | -4.78 |
|  | ND | C | -5.94 |

Supplementary table 7. Connecting letters report from the Tukey HSD multiple comparisons test on how diet effected the parameters measured on honey bees at the end of the experiment. The diet order is arranged from the highest to lowest and the least squares means is on the transformed data. Different letters represent significant differences. Asterisks denote a significant effect at *p*<0.05.
